# A study protocol: a community pharmacy-based intervention for improving the management of sleep disorders in the community settings

**DOI:** 10.1186/1472-6963-14-74

**Published:** 2014-02-18

**Authors:** Zaswiza Mohamad Noor, Alesha J Smith, Simon S Smith, Lisa M Nissen

**Affiliations:** 1 School of Pharmacy, Pharmacy Australia Centre of Excellence (PACE), The University of Queensland, 20 Cornwall Street, Woolloongabba, QLD 4102, Australia; 2 Institute for Health and Biomedical Innovation (IHBI) and Centre of Accident Research and Road Safety (CARRSQ), Queensland University of Technology, 130 Victoria Park Road, Kelvin Grove, QLD 4059, Australia; 3 School Clinical Sciences, Faculty of Health, Queensland University of Technology, 130 Victoria Park Road, Kelvin Grove, QLD 4059, Australia; 4 Kulliyyah (Faculty) of Pharmacy, International Islamic University Malaysia, Jalan Sultan Ahmad Shah, Bandar Indera Mahkota, Kuantan, Pahang 25200, Malaysia; 5 School of Pharmacy, University of Otago, North Dunedin, Dunedin 9016, New Zealand

**Keywords:** Community pharmacy, Actigraphy, Sleep disorders

## Abstract

**Background:**

Sleep disorders are very common in the community and are estimated to affect up to 45% of the world’s population. Pharmacists are in a position to give advice and provide appropriate services to individuals who are unable to easily access medical care. The purpose of this study is to develop an intervention to improve the management of sleep disorders in the community. The aims are– (1) to evaluate the effectiveness of a community pharmacy-based intervention in managing sleep disorders, (2) to evaluate the role of actigraph as an objective measure in monitoring certain sleep disorders and (3) to evaluate the extended role of community pharmacists in managing sleep disorders. This intervention is developed to monitor individuals undergoing treatment and overcome the difficulties in validating self-reported feedback.

**Method/design:**

This is a community-based intervention, prospective, controlled trial, with one intervention group and one control group, comparing individuals receiving a structured intervention with those receiving usual care for sleep-related disorders at community pharmacies.

**Discussion:**

This study will demonstrate the utilisation and efficacy of community pharmacy-based intervention to manage sleep disorders in the community, and will assess the possibility of implementing this intervention into the community pharmacy workflow.

**Trial registration:**

Australian New Zealand Clinical Trial Registry: ACTRN12612000825853

## Background

Sleep disorders appear to be a global epidemic, affecting up to 45% of the world’s population [1]. Sleep disorders encompass problems with falling or staying asleep, waking up too early or too late, and problems with poor sleep quality, all of which may cause significant impairment of daytime functioning.

The community pharmacy is accessible and can provide services to patients who may not regularly come into contact with general practitioners or other traditional sources of health care [2]. Community pharmacy can therefore offer primary assistance for sleep-related disorders to the community. Even though specialist sleep clinics are available, they may not be accessible everywhere especially in rural and remote areas. These specialist clinics tend to focus very much on respiratory disorders of sleep and are considered as tertiary centres which treat later stage or more severe sleep disorders only. Community pharmacists are already in a suitable position to initiate conversation, discuss medicines, and provide ongoing follow-up [3] related to a range of health problems, and many interventions have been implemented for other chronic health problems such as asthma [4-8], diabetes [9-11] and hypertension [12,13]. There are many similarities between these chronic health problems and sleep disorders in terms of early intervention, community-level management and continuing care provision. This suggests that pharmacy-based management of sleep disorders could have a significant role in effectively reducing the burden of these disorders.

There are many types of sleep disorders, but by far the most prevalent in adults are insomnia and obstructive sleep apnoea (OSA) [14-16]. The current gold standard treatments for diagnosed OSA and insomnia patients are continuous positive airway pressure (CPAP) [17,18] and cognitive behavioural therapy (CBTi) respectively [19,20]. However, due to varying results or personal preference [21], many of these patients still present at the community pharmacy seeking additional or alternative treatment options [22,23]. Pharmacists also assist many ‘walk-in’ individuals who often use the community pharmacy as a primary contact to seek help for a variety of problems including sleep-related disorders. Many of these individuals have common or overlapping symptoms or simply present with ‘poor sleep’, thus make it difficult for health professionals to accurately assess and/or treat.

Currently, in managing sleep-related disorders, community pharmacists depend on self-report from patients or on sleep diaries, if indicated. Monitoring sleep disorders is difficult without appropriate tools, education or standard measures. Determining the actual sleep problem and planning meaningful strategies can therefore be complicated without objective measures. To date, in our knowledge, most studies involving sleep disorders in community pharmacies focused on screening tools [24-26] with very few studies incorporating patient follow-up and monitoring by community pharmacists [27].

This study will use a step-wise approach designed to tackle ‘poor sleep’ in all individuals with disturbed sleep, with referral procedures in place if the problem is not resolved. We will investigate the potential extended role of the pharmacists and the development of an intervention to monitor individuals with poor sleep using a convenient and portable measuring device as an objective measure to gain feedback on sleep. A wrist-actigraph (SBV2 Readiband™) allows pharmacy staff to follow those undergoing treatment by measuring the quality and quantity of sleep, and to better characterise their sleep. While monitoring treatment is difficult if self-reporting is poor, the use of actigraphy can overcomes this challenge by providing objective, patient friendly and graphical feedback that confirms certain sleep parameters and validates the self-reported feedback. An individual actigraphy report can be used as the focus reference in patients’ consultations regarding sleep.

As a portable device, the actigraph can record movement over extended periods of time, and has been used extensively in the studies of sleep and circadian rhythm [28-34]. Guidelines by the American Academy of Sleep Medicine (AASM) signified that actigraphy is a very useful method but it is not sufficient for all diagnoses. Clinical guidelines and research suggested that actigraphy is particularly useful in the evaluation of insomnia, circadian rhythm sleep disorders (e.g. advance sleep-phase syndrome (ASPS), delayed sleep-phase syndrome (DSPS), jet lag and shift work sleep disorder), sleep related breathing disorders (e.g. sleep apnoea), determination of response to treatment and in an evaluation of sleep patterns in special populations [28,29]. The actigraph can be used at home by patients without supervision from a trained sleep technician. Positive feedback was received in a feasibility study to determine the possibility and acceptability of actigraphy as a home-base sleep measure. Participants admitted no disruptions to daily tasks while wearing it [35].

This paper describes the research protocol of our study, developed as a community pharmacy-based intervention to improve the management of sleep disorders in the community.

### Research aims

The purpose of this study is to develop an intervention to improve the management of sleep disorders in the community. The aims are: (1) to evaluate the effectiveness of a community pharmacy-based intervention in managing sleep disorders, (2) to evaluate the role of actigraph as an objective measure in monitoring certain sleep disorders and (3) to evaluate the extended role of community pharmacists in managing sleep disorders.

## Methods and design

### Study design

**Figure 1:**
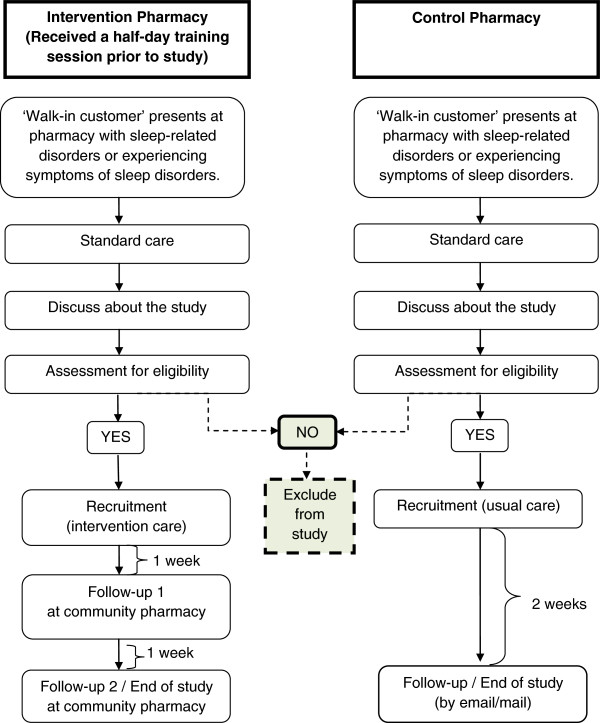
Study design (File attached).

This is a community-based intervention, prospective, controlled trial, with one intervention group and one control group (Figure 1).

### Study setting

Four to five community pharmacies in the Brisbane metropolitan area with similar demographic criteria and physical locations will be recruited based on convenience sampling.

### Ethics approval

This project has been approved by the School of Pharmacy Ethics Committee, The University of Queensland (Reference number: 2012/04).

### Pharmacies

#### *Pharmacy eligibility criteria*

Community pharmacies that meet the following criteria are eligible to participate as a study site:

• Pharmacies that have high daily ‘walk-in customer’ turnover i.e. 100 to 300 customers per day for any ‘Pharmacist only medicine’, ‘Pharmacy medicine’, over-the-counter medicines including herbal supplements.

Additional criteria for the ‘Intervention care group’ pharmacies:

• Agree to install a software application (Sleep Consultant™ by Fatigue Science) which enables data to be downloaded from the actigraph to generate an individual sleep report for the participants.

• Have a private counselling area within the pharmacy where one-to-one consultations with customers will be separated from the common pharmacy counter.

• Able to follow-up participants for 2 weeks from baseline.

#### *Recruitment of pharmacies*

Contact information of the pharmacies will be obtained from the list of community pharmacies from publically available lists. Community pharmacies which meet the inclusion criteria will be invited to join the study by telephone and informed about the project. If the pharmacist expresses an interest, a research officer will arrange a face-to-face discussion for further explanation of the study and obtain consent. Upon agreement, the pharmacies will be assigned to either intervention or control group, based on convenience sampling.

#### *Training for pharmacists and pharmacy assistants*

This study will involve both pharmacists and pharmacy assistants. One survey found that only 30% of direct sleep product requests were handled entirely by pharmacists and in a symptom based scenario, only 18% of requests were handled by pharmacists [36]. Thus pharmacy assistants will be included to reflect common practice and support the pharmacists.

Pharmacists and pharmacy assistants from the intervention pharmacies will attend a half-day training course provided by a sleep psychologist focusing on the use of the actigraph and sleep diaries, sleep scale scores assessment and questionnaires, and provide information on a healthy lifestyle and sleep-related disorders. The actigraph user’s manual will be provided.

### Participants

#### *Sample size*

In similar population-based studies which correlate the results of actigraphy against other measures, i.e. polysomnography and/or sleep diaries, the number of participants recruited varies, ranging from 30 to 450 participants [37-39]. To our knowledge, there is no published research relating to community pharmacy-based interventions utilising actigraphy in sleep health management, therefore no standard reference could be used to determine sample size required for this study. In this study, sample size calculation is based on differences in sleep parameters (sleep onset latency (SOL) and total sleep time (TST)) between actigraphy and sleep diaries [40,41]. Using Power and Sample Size Calculation software version 3.0 2009 (Vanderbilt University), to demonstrate a 20% difference in those sleep parameters between actigraphy versus sleep diary, with 80% power sample and a *p*-value of 0.05, 25 participants are required per study group. To allow for potential dropouts (approximately 10% over 2 weeks), a minimum of 55 participants will be recruited.

#### *Recruitment of participants*

Potential participants will be selected from walk-in customers who present at the participating pharmacies seeking help for sleep-related disorders or having symptoms of sleep problems. In both the intervention and control groups, in addition to the usual discussion and service about sleep health, the pharmacist or the pharmacy staff will invite potential participants to join the study. If they show an interest, further discussion will be provided at a private area in the pharmacy prior to obtaining consent. Those who agree to join the study, will be assigned to either intervention or control group participants, based on which pharmacy they attend.

#### *Intervention participants - intervention care group (ICG)*

At baseline, study eligibility (Table 1) will be checked and inform consent will be obtained before the recruited participants complete the ‘Initial Pharmacy Visit’ questionnaires. Sleep-hygiene advice will also be provided to the participants. Due to the pragmatic nature of the study, eligibility criteria are not restricted to specific sleep disorders.

**Table 1:** Participant eligibility criteria

**Eligibility criteria**	
**Inclusion**	**Exclusion**
• Aged ≥ 18 years old	• Aged < 18 years old
• Attend a participating pharmacy as a ‘walk-in customer’ seeking help for sleep-related disorders or having symptoms of sleep disorders.	• Not able to speak, read and write in English, or not fluent in English and cannot arrange for a translator themselves or a translator is not available.
	• Unable to complete the screening (in the pharmacist’s opinion).
	• Refuse to give consent.
	• Pregnant.
	• Currently under treatment with continuous positive airway pressure (CPAP)

The actigraph is a useful device for monitoring sleep-related disorders, but literature suggests that it should be used in conjunction with other parameters, such as sleep diaries [31,42] as both may complement each other by providing objective and subjective data, respectively. Therefore, participants will be provided with an intervention package, which includes:

1. An actigraph (SBV2 Readiband™) to be worn 24-hours a day for seven days before participants revisit the pharmacy for their first follow-up, and then for a further seven days after the first follow-up before returning to the pharmacy for final assessment.

2. A sleep diary [43] to self-record 14 days of bedtime, wake time, time to fall asleep, number of nocturnal awakenings and total sleep time, plus information related to sleep-hygiene, lifestyle and other factors that may interrupt sleep.

3. Educational information about sleep disorders, sleep health management, sleep hygiene and healthy lifestyle to improve sleep (via access link to a website: http://sleepproblemandyou.wordpress.com/ which is developed specifically for this study). The web address will be provided to each participants.

One complete study duration for a participant in the intervention group is two weeks with follow-ups at two time points; week 1 (day 8) and week 2 (day 15). At both time points, pharmacy staff will analyse the data downloaded from the actigraph using Sleep Consultant™ software to generate an on-the-spot individualise report of sleep/wake patterns and they will also assess the self-report information from the sleep diary.

##### 

**Follow-up 1** The participants will receive sleep advice and possible solutions focusing on behavioural strategies such as sleep hygiene, change of lifestyle, daily alcohol and caffeinated drinks consumption and activities before going to sleep, based on data gained from both parameters. Any requests for sleep medicine (‘Pharmacist only medicine’ or ‘Pharmacy medicine’) will follow the pharmacy’s standard practice. After this session, the participants will continue to wear the actigraph plus complete the sleep diary for another seven days.

##### 

**Follow-up 2 / end of study** Participants’ first (pre) and second (post) individual sleep reports will be compared to assess any particular improvement in their sleep condition after receiving sleep advice and possible solution during the first follow-up. Participants will also complete the ‘End of Study’ questionnaires. In certain circumstances (see the ‘Protocol to refer participant to a general practitioner’ subsection), pharmacists can recommend a GP referral for further examination.

##### 

**Protocol to refer participant to a general practitioner (GP) at the end of study** In these circumstances: (1) if the sleep efficiency percentage (SE%) is below 85% at the end of study (normal SE% for adult if measure using actigraphy is 85% and above) [41,44], and (2) if analysis of the data collected appears to indicate that he/she may be at risk of having/developing sleep-related problems and has reported symptoms such as:

• choking or suffocating during sleep

• stopping breathing when sleep

• snoring during sleep

• excessive daytime sleepiness

• difficulty falling asleep at night

• having problems waking up in the morning

• falling asleep too early at night

• waking up too early at night

• fatigue and having difficulty concentrating on daily tasks

• experiencing unpleasant sensations with an urge to move their limbs

Above symptoms may be indicative of another sleep disorders, specifically OSA, narcolepsy, a circadian rhythm disorder, restless legs syndromes or a primary mood disorder.

#### *Control participants - usual care group (UCG)*

The usual care group (UCG) participants will receive standard or usual care for sleep disorders based on the usual practice in the community pharmacy. Australian community pharmacies usually follow the Pharmaceutical Society of Australia’s recommended practice [45], which includes supplying ‘Pharmacy medicine’, ‘Pharmacist only medicine’, complementary medicine or other over-the-counter (OTC) medicines for sleep-related disorders, if indicated. At baseline, follow the same protocol as in the intervention group, study eligibility (Table 1) will be checked and inform consent will be obtained before the participants complete the ‘Initial Pharmacy Visit’ questionnaires. Baseline demographic and assessments will be completed for comparisons with the ICG participants. The UCG participants will be followed-up after two weeks by the study researcher via email or mail to complete the ‘End of Study’ questionnaires.

### Study measurements and outcomes

#### *Study measurements*

Assessments will be conducted at three times points – baseline, week 1 and week 2 (Table 2). At baseline upon recruitment, participants will be assisted to complete the self-administered ‘Initial Pharmacy Visit’ questionnaires, comprise of:

• Demographic and lifestyle information, which includes sleep environment, smoking, alcohol consumption and caffeinated drinks intake, modified from the validated ‘Pharmacy Tool for Assessment of Sleep Health - POTASH’ [24].

• Health-related quality of life (HRQOL) assessment using validated WHO-Five Well Being Index (version 1998) [46,47].

• Sleep health assessment using a set of survey instruments adapted from POTASH [24]: Epworth Sleepiness Scale (ESS) [48,49], Insomnia Severity Index (ISI) [50], Multivariate Apnea Prediction Index (MAPI) [51] and International Restless Legs Syndrome Study Group (IRLSSG) [52].

• Community pharmacy survey: 14 questions using a 5-point Likert-type scale from 1 (strongly disagree) to 5 (strongly agree), and one open ended question.

**Table 2:** Summary of measurements and study outcomes

**Measurements**	**Instruments**	**Group**	**T0**	**T1**	**T2**	**Outcome**	
Surveys/measuring tools							
Socio-demographic and lifestyle	Questionnaire	ICG, UCG	x				2˚
Health-related quality of life ^I,E^	WHO-5 Well-being index (1998)	ICG, UCG	x		x	1˚	
Sleep scale scores ^I,E^	ESS, ISI, MAPI, IRLSSG	ICG, UCG	x		x	1˚	
Community pharmacy survey ^I^	Questionnaire	ICG, UCG	x				2˚
Close-out survey ^E^	Questionnaire	ICG, UCG			x	1˚	2˚
Sleep parameters assessment							
SE%	Actigraph only	ICG		x	x	1˚	
TST, NWAK, SOL	Actigraph and sleep	ICG		x	x	1˚	
	diary						
Other factors that may interrupt sleep	Sleep diary only	ICG		x	x	1˚	
Pharmacy survey							
Close-out survey for pharmacy	Questionnaire	Pharmacists, pharmacy staff			x		2˚

T0 = baseline, T1 = 1 week after baseline, T2 = 2 weeks after baseline.

ICG: intervention care group; UCG: usual care group.

ESS: Epworth Sleepiness Scale; ISI: Insomnia Severity Index; MAPI: Multivariate Apnea Prediction Index; IRLSSG: International Restless Legs Syndrome Study Group.

SE%: Percentage of sleep efficiency; TST: Total sleep time per-24 hour period; NWAK: Number of nocturnal awakenings; SOL: Sleep onset latency.

1˚: Primary outcome; 2˚: Secondary outcome.

I: ‘Initial Pharmacy Visit’ Questionnaires; E: ‘End of Study’ Questionnaires.

Follow-up after one week from baseline will be conducted in the ICG only (Table 2). Assessment (pre) of sleep parameters will be obtained from the actigraph and sleep diary for these measures: (i) Sleep efficiency percentage (SE%), (ii) Total sleep time (TST) per 24-hour period (hours/day), (iii) Number of nocturnal awakenings (NWAK), and (iv) Sleep onset latency (SOL).

Follow-up at week 2 will be conducted to complete the study in both groups (Table 2). Assessment (post) of sleep parameters will be obtained from the actigraph and sleep diary, as in previous follow-up. Participants will also complete the ‘End of Study’ questionnaires, comprise of:

• HRQOL assessment using validated WHO-Five Well Being Index (version 1998).

• Sleep health assessment (consist of ESS, ISI, MAPI and IRLSSG), adapted from POTASH [24].

• A close-out survey, consisting of: (i) questionnaire on sleep-related lifestyle and behaviour changes since completing the study, (ii) self-opinion of sleep health after the study, and (iii) willingness and ability to pay if such program is offered as a service by community pharmacy.

Upon completion of the study, pharmacists and pharmacy staff will complete a self-administered questionnaire on the feasibility of the study, and opinions on the management of sleep disorders after participating in the study (Table 2).

#### *Study outcomes*

The primary outcomes (Table 2) of the study will be evaluated based on these three objectives:

1. To evaluate a community pharmacy-based ‘model of care’ to improve the management of sleep disorders in the community, by comparing:

a. Changes in HRQOL mean scores between and within the intervention care group (ICG) and usual care group (UCG) at baseline and week 2.

b. Changes in sleep scale mean scores – ESS, ISI, MAPI and IRLSSG, between and within the intervention care group (ICG) and usual care group (UCG) at baseline and week 2.

c. Changes in sleep parameter mean scores – sleep efficiency percentage (SE%), total sleep time (TST), number of nocturnal awakenings (NWAK) and sleep onset latency (SOL), between pre– and post–actigraphy sleep report data in the intervention care group (ICG).

2. To evaluate the role of actigraph as an objective sleep assessment instrument to monitor certain sleep disorders, by comparing:

a. Changes in sleep parameter mean scores –total sleep time (TST), number of nocturnal awakenings (NWAK) and sleep onset latency (SOL), between actigraphy and sleep diary at week 1 and week 2 in the intervention care group (ICG).

3. To evaluate the extended role of community pharmacists in managing sleep disorders in the community, by evaluating:

a. Participants’ opinions of sleep health at the end of the study.

b. Willingness and the amount able to pay if such programme is provided as a service in the community pharmacy.

Secondary outcomes (Table 2) to be evaluated are:

1. Participants’ opinions of the community pharmacy and opinions on the use of community pharmacy to seek help for sleep-related disorders.

2. Comparison between groups in sleep-related lifestyle and behaviours at week 2 follow-up.

3. Pharmacist and pharmacy staff opinions regarding the feasibility of the study and the management of sleep disorders in the community pharmacy after participating in the study.

### Data analysis

Data will be collected from the sleep report generated from the Sleep Consultant™ software, sleep diary, and questionnaires (initial and end of study). Sleep parameters measurement, i.e. sleep efficiency percentage (SE%), total sleep time (TST), sleep onset latency (SOL) and number of nocturnal awakenings (NWAK) of a single participant through either the sleep diary or actigraphy will be averaged from all the data recorded during the 2-week period (recorded as ‘pre’ (week 1) and ‘post’ (week 2) mean scores). There will be no discrimination between weekdays and weekend data. Sleep scale scores to assess sleep health using ESS, ISI, MAPI and IRLSSG survey recorded at baseline and week 2 will be averaged to mean scores for analysis.

Data will be analysed using SPSS 20.0 for windows [53]. This study will consider alpha level of 0.05 for all statistical tests. Using descriptive, paired *t*-test and independent *t*-test – demographic characteristics and sleep scale scores between and within groups will be compared. In the ICG, pairwise differences of sleep parameters mean scores between actigraphy and sleep diary will be calculated using independent *t*-test, and will be used paired-*t*-test in comparing within group, for pre– and post–mean scores (for normally distributed data). To determine correlation between two variables, Pearson’s correlation will be applied. Likert-type questions will be analysed as descriptive analysis.

## Discussion

Sleep disorders are a concern worldwide and an emerging public health problem [1,54,55], thus it requires a range of strategies from public education through to clinical services to manage it. Exploring and developing new interventions to improve the management of sleep disorders within the primary healthcare system such as a community pharmacy-based approach is crucial as health care costs continue to increase [56]. Pharmacists are in a suitable position to provide an appropriate and vital step to improve sleep health management [24-27]. To our knowledge, this is the first community pharmacy-based study evaluating an intervention integrated into the community pharmacy workflow, to enable pharmacists to improve the management of sleep disorders.

## Abbreviations

AASM: American academy of sleep medicine; ASPS: Advance sleep-phase syndrome; CBTi: Cognitive behavioural therapy for insomnia; CPAP: Continuous positive airway pressure; DSPS: Delayed sleep-phase syndrome; ESS: Epworth sleepiness scale; HRQOL: Health-related quality of life; ICG: Intervention care group; IRLSSG: International restless legs syndrome study group; ISI: Insomnia severity index; MAPI: Multivariate apnea prediction index; NWAK: Number of nocturnal awakenings; OSA: Obstructive sleep apnoea; SOL: Sleep onset latency; SE: Sleep efficiency; TST: Total sleep time; UCG: Usual care group.

## Competing interests

The authors declare that they have no competing interests.

## Authors’ contributions

ZMN is lead investigator and wrote the first draft with input from AS, SS and LS. The study design was done by ZMN and AS. All authors have revised and corrected draft versions and approved for the final version of the manuscript.

## Pre-publication history

The pre-publication history for this paper can be accessed here:

http://www.biomedcentral.com/1472-6963/14/74/prepub
